# The Political Economy of Aging Services in the United States Since the Aging Enterprise

**DOI:** 10.1093/geroni/igaa057.2538

**Published:** 2020-12-16

**Authors:** Larry Polivka

**Affiliations:** Florida State University, Tallahassee, Florida, United States

## Abstract

The Aging Enterprise was the first book length analysis of the origins and operations of the aging services system that grew out of the 1963 Older Americans Act and provided the first state system of community-based services. The book also featured an original critique of the aging services system based on an application of the theoretical perspectives that were new to social gerontology. This presentation will use one of these perspectives, the political economy of aging in the U.S., to chart changes in aging services systems since the publication of The Aging Enterprise. The presentation will show that Estes’ pioneering work on the application of the political economy perspective on aging policies and programs in the US, is more relevant to our understanding of these programs than it was 40 years ago, by describing and analyzing the increasing control of for profit corporations over the aging services systems. Part of a symposium sponsored by the Women's Issues Interest Group.

